# The Role of Microbiota in Upper and Lower Gastrointestinal Functional Disorders

**DOI:** 10.3390/microorganisms11040980

**Published:** 2023-04-09

**Authors:** Francesco Vito Mandarino, Emanuele Sinagra, Dario Raimondo, Silvio Danese

**Affiliations:** 1 Division of Gastroenterology and Gastrointestinal Endoscopy, San Raffaele Hospital, 20132 Milan, Italy; 2 Gastroenterology & Endoscopy Unit, Fondazione Istituto G. Giglio, Contrada Pietra Pollastra Pisciotto, 90015 Cefalù, Italy

Functional gastrointestinal disorders (FGIDs), also known as disorders of gut–brain interaction, are a group of disorders characterized by chronic gastrointestinal symptoms in the absence of demonstrable pathology on conventional testing. They are commonly encountered in clinical practice and the community [1]; according to a recent survey that utilized the Rome IV diagnostic criteria, the worldwide prevalence of FGIDs is approximately 40% [2].

The most recent classification scheme (ROME IV) categorizes FGIDs into 33 adult disorders and 20 paediatric disorders; the most prevalent subtypes are irritable bowel syndrome (IBS), which is characterized by abdominal discomfort, altered bowel habits, and bloating, and functional dyspepsia (FD), which causes epigastric pain, discomfort, and satiety [3].

While the pathophysiology of FGIDs is complex, it has been reported that intestinal microbiota harbours a pivotal role in both the development of FGID and the modulation of clinical symptoms [4,5]. Remarkably, the latest evidence has shown that FGIDs [6,7] are linked with intestinal dysbiosis, defined as relevant changes in the diversity, density, or metabolic activity of gut bacteria [8].

Previously, FGIDs were primarily linked with psychosocial conditions, but an improved understanding of their pathophysiology has changed this perception. The management of patients with FGIDs now considers their genetic predisposition, epigenetics, neural connections, lifestyle habits, enteric nervous system (ENS), environmental factors, and their interaction with microbiota [3]. It must be said that recently there have been significant advances in the available therapeutic options for FGIDs, including even endoscopic treatment options, such as in cases of refractory gastroparesis [9,10].

The gut microbiome is a heterogeneously dense microbial system that regulates the physiology and pathophysiology of the host through a broad network of biochemical pathways [3,11].

Initially, the pathogenic component of gut microbiota was shown to be associated with FGIDs, with up to 10% of irritable bowel syndrome (IBS) patients having gastrointestinal infections followed by gut microbiota dysbiosis, resulting in the occurrence of IBS (postinfectious IBS) [12].

Advances in technology have improved our understanding of the gut microbiome [13].

One of the compelling pieces of evidence of the role of microbiota in FGIDs originates from studies in germ-free (GF) mice, which showed alterations in gut transit, visceral sensation, and intestinal barrier function after faecal microbiota transplantation (FMT) from patients with FGIDs [3,14,15].

Changes in gut microbiota in the small bowel and colon have been extensively studied in patients with FGIDs [3,12], as they can affect gut motility, intestinal gas profile, gut immune and intestinal barrier function, visceral sensation, neuro-immuno-endocrine interface and, finally, gut–brain axis [3,16,17].

Remarkably, dysregulation of the gut–brain axis is a primary mechanism in some FGIDs patients [3,18], where the interplay between the gut and brain is critical [19,20]: the brain modifies gut physiology, such as visceral sensitivity and motility, thus causing symptoms of FGIDs [21], while changes in the gut can impact psychological well-being [22].

Gut microbiota dysbiosis can be modified by various treatments; these changes may be understood as a trigger for FGIDs, or they may be the goal of the treatment, impacting symptoms.

Dietary intervention represents the simplest therapy to restore healthy gut microbiota, such as consuming fiber-rich diets to increase the production of short-chain fatty acids or following a low-FODMAP diet [3].

Probiotics have been demonstrated to be effective in alleviating FGIDs symptoms; however, more evidence is needed [3].

Antibiotics have been suggested to play a pivotal role in the development of IBS [23]. Several studies have shown that antibacterial therapy induces shifts in bacterial community composition such as those seen in IBS. This finding is further suggested by data from cohort and case-control studies that have highlighted an increased risk of IBS associated with antibiotic treatment. On the other hand, rifaximin has shown benefits in non-C-IBS [24], and there is optimism regarding its use in C-IBS [25,26].

The use of FMT is believed to be helpful in treating gut dysbiosis by restoring a “healthy” microbial environment. However, it is still unclear whether FMT is an effective therapy for FGIDs or whether it only works as a placebo [27,28,29].

The characterization of gut microbiota species in healthy and FGID patients has made significant progress. However, further studies in human patients and animal models are needed to ensure the exact roles of microbiota and its derived metabolites that are connected to the pathophysiological pathways of FGIDs. Furthermore, new data will clarify ways in which restoring healthy gut microbiota would keep the microbiota–gut–brain axis in balance and improve clinical symptoms.

Beyond FGIDs, gut microbiota also plays a critical role in the pathogenesis of organic diseases of the digestive tract, such as inflammatory bowel diseases [30,31,32], eosinophilic esophagitis [33], and others [34], as well as in the development of post-surgical complications [35,36], which require endoscopic treatment [37,38,39,40,41]. Characterization of the microbiota may represent a future therapeutic approach for these conditions as well.

In this Special Issue of *Microorganisms*, state-of-the-art studies that emphasize the role of the gut microbiome in influencing the main pathophysiological mechanisms involved in FGIDs are welcomed. Our goal is to implement knowledge of ways in which characterization and study of the microbiota may contribute to FGIDs, from pathophysiological aspects to new therapeutic applications.
